# Immediate impact of stay-at-home orders to control COVID-19 transmission on mental well-being in Bangladeshi adults: Patterns, Explanations, and future directions

**DOI:** 10.1186/s13104-020-05345-2

**Published:** 2020-10-22

**Authors:** Mohammad Ali, Gias U. Ahsan, Risliana Khan, Hasinur Rahman Khan, Ahmed Hossain

**Affiliations:** 1 Department of Physiotherapy and Rehabilitation, Uttara Adhunik Medical College and Hospital, Uttara, Dhaka, 1230 Bangladesh; 2 Department of Public Health, North South University, Bashundhara, Dhaka, 1229 Bangladesh; 3 NSU Global Health Institute, North South University, Dhaka, 1229 Bangladesh; 4 Health Management BD Foundation, Dhaka, Bangladesh; 5 International Dhaka School, Dhaka, Bangladesh; 6 Institute of Statistical Research and Training, Dhaka University, Dhaka, Bangladesh

**Keywords:** Bangladesh, Covid-19 pandemic, Depression, Lockdown, Mental well-being, Social distance

## Abstract

**Objective:**

We aim to evaluate the immediate impacts of COVID-19 stay-at-home orders on the mental well-being of Bangladeshi adults. We recruited 1404 healthy adults following the Bangladesh government's lockdown announcement. A questionnaire comprising the Warwick Edinburgh Mental Well-being Scale was used to define mental health.

**Results:**

The overall mean score for well-being was 42.4, indicating that 51.9% of adults suffered from poor mental health. And within that 48% of males and 57% of females were depressed. The mean scores for government workers, unemployed workers, and business employees were 45.1, 39.6, and 39.5, respectively. Confounding adjustments in multivariable linear regression models revealed that married women, unemployed and business communities, and individuals returning to villages were heavily depressed. Stay-at-home orders had significant repercussions on mental health and created a gender disparity in depression among adults. Suggestions include promoting mental health for women, unemployed, and business individuals. Married women need to be taken into special consideration as their mental well-being is worse. Older people (50 years of age and over) reported a high day-to-day variation in their mental health. These results should be factored in when discussing the mental health of adults and communities to cope with quarantine.

## Introduction

The world never encountered the phenomenon of social distancing or isolation on such a scale before the coronavirus pandemic in 2019 (COVID-19). The World Health Organization’s recommended ‘quarantine’ has proven to be an effective method of minimizing the spread of the virus [1]. The Bangladeshi government issued stay-at-home orders on March 26 as a public health invention to bring down perpetual transmissions rates [2]. This led to the lockdown of business centers, governmental offices, and non-government workplaces [3]. Individuals are plagued with the fear of contracting COVID-19 and uncertainties due to forced cohabitation, which in turn adversely affects human psychology and mental health [4]. Studies showed a strong connection between quarantine and cognitive function, neuroendocrine, and cardiovascular systems, which can lead to depression, cognitive impairment, and sleep disturbance [5, 6]. Besides, quarantine creates poor behavioral patterns through substance abuse, crime, and even suicide [7, 8]. Bangladesh already reported its first quarantine related suicide case [9]. Psychological effects include post-traumatic stress, frustration, and rage [10]. For the aforementioned reasons, mental well-being data during the lockdown period is crucial for policymakers to assess pre and post-pandemic life.

The lack of positive mental well-being holds a significant determinant of physical health and longevity [7–9]. When one is feeling good' and 'functioning well' they are simultaneously optimistic, happy, resilient, have higher self-esteem and agency, and indulge in good relationships [7–14]. Mental well-being has also been found to be associated with better occupational functioning [1, 2, 15]. This means the absence of positive mental well-being causes depression.

The immediate effect of lockdowns in Bangladesh on individuals and communities is incomprehensible. Although quarantine may curb the spread of the virus, with millions of people confined at home long-term irreversible psychiatric disorders with negative social implications may be widespread. Preventing emotional and behavioral issues and encouraging physical, social, and mental well-being should be priorities for Bangladesh. By the same token, safety nets may be limited or absent to protect incomes and livelihoods [16]. Thus, we aimed to assess the immediate impact of the lockdown on mental well-being in Bangladesh.

## Main text

### Methods

#### Study population

We conducted a cross-sectional study to measure the association between sociodemographic factors and mental well-being. Bangladeshi citizens were invited to participate in a study via social media during the onset of quarantine. Approximately 2500 people received the invitation from March 28 to April 7, 2020. 1523 participated, giving a 61% overall response rate. 1404 responses were considered for analysis after distinguishing them based on inclusion and exclusion criteria. Inclusion criteria include healthy adults who are between the ages of 16 to 60 years old. Pregnant women and participants having chronic illness were excluded. This research project was approved by the Institutional Review Board (IRB) of North South University (NSU-IRB 3572).

#### Independent variables

The questionnaire included demographic variables, such as age, gender, marital status, current living location, occupation, current working condition, and education status. Current working conditions were classified as not working, working from home, both working from home and in-person to office. The occupational groups were split into business, government, healthcare, housewife/unemployed, non-government, and students.

#### Outcome measure: Warwick Edinburgh Mental Well-being Scale

The Warwick-Edinburgh Mental Well-being Scale (WEMWBS) was used throughout as it provided an appropriate measure of mental well-being. WEMWBS was found to be easy to complete, clear, and unambiguous; it is also popular with practitioners and policymakers [12, 16]. The WEMWBS measures subjectively perceived wellness, and psychological function on a 5-point Likert scale over 2 weeks (14–70 points, a higher score means better mental health). As the scale was not invented through mental illness screening methods, there is no cut-off point [10]. The questionnaire was used in various studies and validated in several languages and settings [11, 13, 14]. Before beginning the survey a user license for using WEMWBS was obtained (Registration ID: 517150559).

#### Statistical analysis

We analyzed data using a software R. The R package ggplot2 was used for graphical presentation. Where an item in the WEMWBS is absent, it substitutes for the mean of other items in the domain. Otherwise, we deleted the participant from the analysis with the missingness of two or more than two items in the WEMWBS items. Sociodemographic characteristics were described by frequency (percent), mean, and standard deviation. An independent t-test, and one-way ANOVA, were used to determine the relationship between socio-demographic factors and well-being scores. Afterward, a multivariable linear regression model was used to control confounding variables (Model 1) using the lm() function in R. A separate multivariable linear regression model was generated after adjusting the confounding variables to stratify the analysis by male and female groups (Model 2 and Model 3). Factors at the 5% significance level were defined as statistically significant. We evaluated the internal consistency of WEMWBS using Cronbach's alpha [17].

### Results

#### Sociodemographic characteristics

Table 1 shows the sociodemographic characteristics in comparison to the WEMWBS ratings. Of the 1404 respondents, 63.2% were male. 83% of subjects were with undergraduate degrees or above. The mean score for WEMWBS was 42.5 (standard deviation, SD 11.1), and the median was 42. For men, the mean change of well-being score was 1.7 points, which is higher than that of women. Thus, elucidating those female participants may suffer from worse mental health. Cronbach's alpha for WEMWBS was 0.88 (95% CI [0.85; 0.90]), which indicates an excellent internal consistency. A significant association was found between WEMWBS scores and occupation. Variabilities were more prominent in age groups 16–19 and older than 50 years, which indicates that those age groups may face psychological instability.

**Table 1:** Participant characteristics and association with wellbeing scores

Variables	Categories	n	Mean (sd)	P-value*
Age group	16–19	50 (3.6%)	42.74 (12.78)	0.087
	20–29	767 (54.6%)	42.06 (10.85)	
	30–39	447 (31.8%)	42.24 (10.35)	
	40–49	110 (7.8%)	45.11 (10.89)	
	≥ 50	30 (2.1%)	44.06 (12.65)	
Gender*	Male	888 (63.2%)	43.04 (10.82)	0.007
	Female	516 (36.8%)	41.37 (11.22)	
Marital Status	Married	713 (50.8%)	42.29 (10.97)	0.799
	Never-Married	671 (47.8)	42.60 (10.91)	
	Others	20 (1.4%)	41.35 (12.63)	
Education	Schooling 6–12 years	232 (16.5%)	42.03 (11.18)	0.777
	Undergraduate	576 (41.0%)	42.37 (10.83)	
	Graduate	596 (42.5%)	42.63 (11.18)	
Occupation	Business	79 (5.6%)	39.53 (11.42)	< 0.001
	Government	69 (4.9%)	45.14 (11.79)	
	Healthcare	356 (25.4%)	43.62 (09.99)	
	Housewife/ Unemployed	129 (9.2%)	39.57 (10.40)	
	Non-government	353 (25.1%)	42.77 (11.66)	
	Student	418 (29.80)	42.10 (11.31)	
Working Condition	Not employed	758 (54.0%)	41.94 (11.12)	0.201
	Work from home	422 (30.1%)	42.93 (11.09)	
	Work from both home and Outside	224 (15.9%)	43.13 (11.01)	
Current location of living*	City	1118 (79.1%)	42.68 (11.09)	0.0961
	Village	286 (20.9%)	41.45 (11.12)	

^*^p-value was calculated from ANOVA, and the p-values for gender and current location of living were calculated from t-test

The well-being score of 51.9% of the participants fell in the range of 14–42 representing low levels of mental well-being. 40.2% of the participants scored 43–59 which is middle-range well-being, and the remaining 7.8% were in the range 60–70 suggesting good mental health. From the results, it is evident global stress has had spillover effects on individuals' mental condition.

#### The gender gap in mental well-being

Depression seemed to be heavily skewed towards women, raising agonizing concerns. For instance, 57.2% of female participants were in poor mental health (i.e. WEMWBS score ≤ 42), whereas for males it was at 48.9%.

#### Patterns of well-being by age and occupational groups

**Fig. 1:**
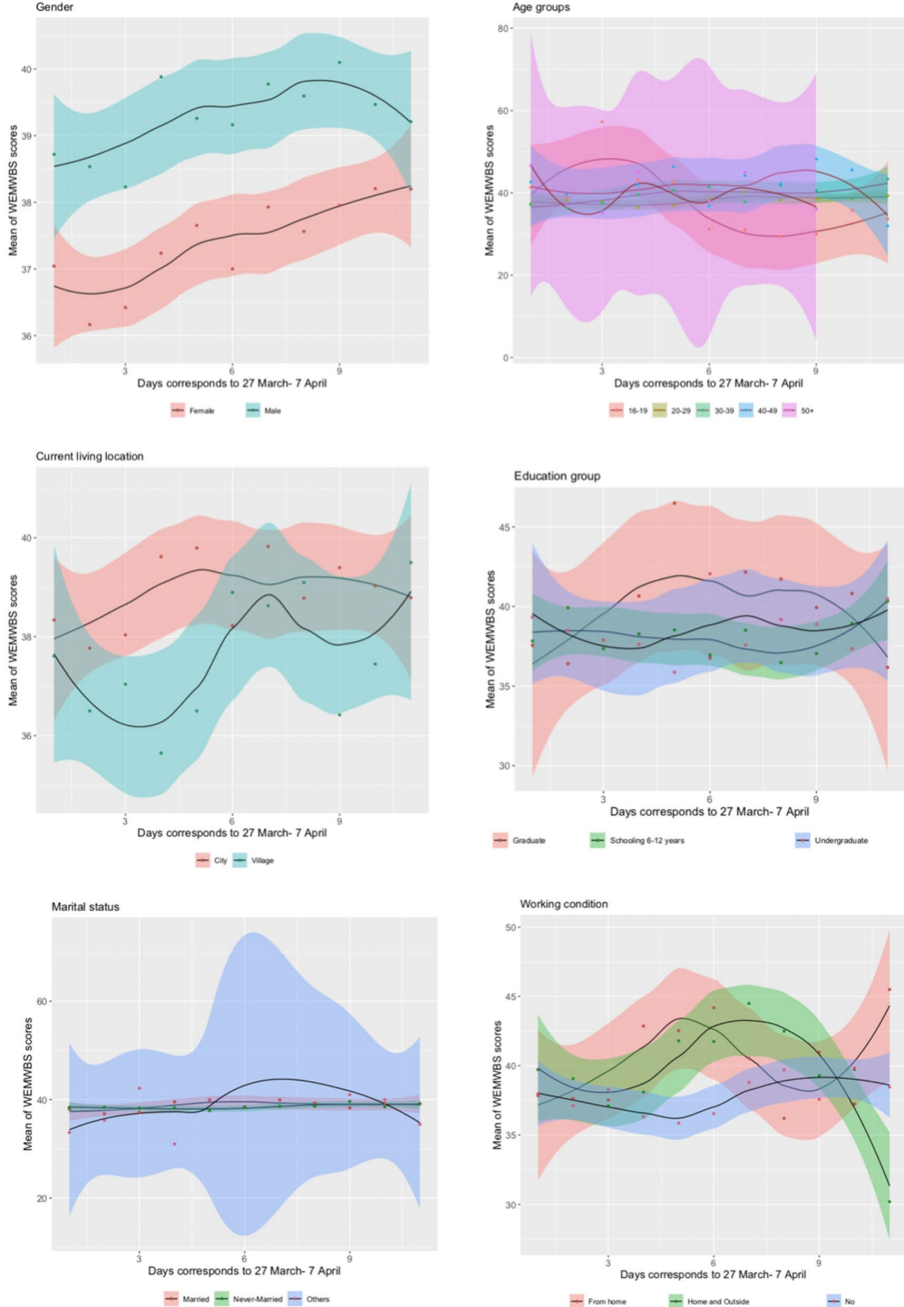
Day-to-day comparison of mental well-being scores

Figure 1 shows a day-to-day comparison of well-being scores at different categories of sociodemographic factors. As days passed the scores for both men and women were seen to increase. The scores for age ≥ 50 had higher variability when compared to other age groups, thereby demonstrating that the elderly population is likely to face far more mental strain. Variations were also observed in the ‘divorced/separated people’ and ‘living in the village’ categories. The participants who were housewives or unemployed fell under the non-working group and were seen to have low WEMWBS scores, but the results remained constant. The participants who worked at home and worked both at home and outside were susceptible to inconsistent data, in other words, the data was highly variable.

#### Multivariable linear regression models

Table 2 presents the results from multivariable linear regression models. The results present the slope estimate, 95% confidence interval of the slope, and the corresponding p-value. We applied three models. The first model used all the data, and the next two models were gender-separated, i.e. women have one model and men have the other. This was done to eliminate confounding bias. From Model 1 it appears that gender and occupational groups have a significant effect on mental well-being scores. When compared to women, men have significantly higher well-being scores with a slope of 1.79 (95% CI = 0.5 to 3.1). The participants who were involved in business had worse mental health than government employees (decreased by 5.87 units, p = 0.01), health care workers (by 4.98, p ≤ 0.001), and employees of private companies (by 3.31, p = 0.02). Model 2 provides similar results as the data is only about men. Most women are not in the working group, and therefore the occupational factor them. Interestingly, the unmarried females appear to have higher well-being scores than the married women (by 3.31, p = 0.01).

**Table 2:** Results from multivariable linear regression model with wellbeing score as outcome. (The positive slope means better mental wellbeing)

Variable	Categories	Model 1 (All data)*	Model 2 (Male data)*	Model 3 (Female data)*
Age	16–19	– 0.71, (– 6.1, 4.7), 0.80	3.37, (– 3.3, 10.1), 0.32	– 5.98, (– 15.4, 3.5), 0.22
	20–29	– 1.56, (– 5.8, 2.7), 0.48	1.66, (– 3.7, 7.1), 0.55	– 5.12, (– 12.4, 2.2), 0.17
	30–39	– 1.24, (– 4.4, 2.9), 0.56	0.74, (– 4.4, 5.9), 0.78	– 3.64, (– 10.9, 3.6), 0.32
	40–49	1.74, (– 2.7, 6.2), 0.45	3.81, (– 1.7, 9.3), 0.17	– 0.84, (– 8.9, 7.2), 0.84
	≥ 50	Reference		
Gender	Male	1.79, (0.5, 3.1), 0.01	–	–
	Female	Reference		
Occupation	Government	5.86, (2.2, 9.5), 0.01	7.88, (3.6, 12.1), < 0.001	– 0.49, (– 9.7, 8.8), 0.91
	Healthcare	4.98, (2.2, 7.8), < 0.001	4.17, (1.0, 7.3), 0.01	3.24, (– 4.9, 11.4), 0.44
	Housewife/ Unemployed	1.56, (– 1.7, 4.8), 0.34	3.82, (– 0.6, 8.3), 0.09	– 2.00, (– 10.3, 6.3), 0.64
	Private	3.31, (0.6, 6.0), 0.02	3.38, (0.5, 6.3), 0.02	0.98, (– 7.4, 9.3), 0.82
	Student	2.97, (0.1, 5.9), 0.04	4.10, (0.8, 7.4), 0.01	– 1.40, (– 9.8, 7.0), 0.74
	Business	Reference		
Working condition	Not-working	– 0.84, (– 2.2, 0.5), 0.22	– 1.29, (– 3.0, 0.4), 0.14	– 0.21, (– 2.4, 1.9), 0. 85
	Both home and outside	– 0.67, (– 2.5, 1.2), 0.48	– 0.41, (– 2.7, 1.8), 0.72	– 1.53, (– 4.7, 1.7), 0.35
	Work from home	Reference		
Marital status	Never married	1.13, (– 0.4, 2.7), 0.15	– 0.63, (– 2.8, 1.5), 0.56	3.31, (1.0, 5.7), 0.01
	Divorced/ Separated	– 0.02, (– 5.0, 4.9), 0.99	– 7.44, (– 17.5, 2.7), 0.15	2.30, (– 3.4, 7.9), 0.42
	Married	Reference		
Current living location	Staying at Village	– 1.02, (– 2.5, 0.5), 0.19	– 1.10, (– 2.9, 0.7), 0.22	– 1.61, (– 4.8, 1.5), 0.32
	Staying at City	Reference		

^*^The values present slope (95% confidence interval) and p-value

### Discussion

One of the most profound impacts of quarantine has been the loss of income endured by many families. Research demonstrates how a physical loss comes in the form of emotional 'loss,’ which creates a knock-on effect, such as diminishing self-worth and motivation. This could be one of the reasons as to why residents in Bangladesh had significantly lower mental well-being scores relative to those for other countries. We used a Bangla version of WEMWBS to define mental well-being, and in the population group, we found a strong internal consistency with a high Cronbach alpha.

Contrary to the study by Murray et al., in which women had significantly higher mental well-being than men [18], we found that in conjunction with high levels of stress, females had poorer mental well-being scores compared to males. Other studies showed similar results. One in China indicated that during the pandemic the mental health of female health care workers took a substantial toll, with them being far more anxious than men [18, 19]. While other research supported the idea that females had lower mental well-being scores throughout the pandemic [20, 21].

Our results reflect more day-to-day variation in the mental well-being scores for age group 50 and above. According to the World Health Organization 2017 report, psychological disorders such as dementia and depression affect 15% of people over 60. So one of the reasons the variability in data could be those elderly individuals are susceptible to depression, which is further heightened by being contained in the house for long periods.

As mentioned, governmental jobs are found to be associated with higher mental health (an increase of 1.6 units) compared to people in businesses. One explanation for that could be financial certainty. Retrospective research indicates a strong link between financial solvency and good mental well-being [22]. Similar to our results, a report found that business and career uncertainty have a substantial adverse effect on the mental health of the adult population [23].

Being single was associated with increased well-being for female participants (an increase of 2.0 units). In Bangladesh, the culture is such that married women face household burdens along with their issues, which means less private time. Putting children and joint families in the mix the household work will exponentially increase. One research concluded that increased household load causes mental illnesses [24]. The pandemic in itself is characterized as a period of distress and difficulty but for married women, their responsibilities are further exacerbated [25–27].

#### Future directions

From the results, it's evident that Bangladeshi women need to have healthy strategies solely implemented towards them, along with psychosocial support. These programs will not only positively impact the target group but will also have spillover benefits for their families. It is worth noting that currently there is no strategic guideline in Bangladesh’s health system for the improvement of mental health practices of adults as a whole. Therefore, it is hoped that the results obtained can showcase the dire need to improve health facilities, especially during the lockdown period. Future studies should evaluate the associated factors explaining a higher prevalence of poor mental health among adults in Bangladesh.

### Conclusion

In this population-based analysis, we found that half of the sample face poor mental health during quarantine, which leads to increased feelings of depression. Mental well-being in the population was worse for women, unemployed people, the business community, and married persons. Staying-at-home not only induces depression among women but also entails a host of other social and economic problems in their families and communities. Older people reported a high day-to-day variation in their mental well-being ratings. Late support could potentially have devastating and lasting impacts on mental health, particularly among the groups that are already socially and economically vulnerable. Moreover, the current trend of depression prevalence impacts all occupational groups. Screening and implementing interventions for depression have been associated with modest long-term impacts for both the family and community. We hope this study forms a basis for practitioners and policymakers to suggest where interventions are necessary to promote mental health.

## Limitations of this study

Firstly, we were unable to provide effects of family income, food security, dietary habits, and physical activity as online survey constraints limited the type of data that could be collected. Second, while the study's cross-sectional nature helped us to explore the associations between mental well-being and socio-demographic factors, it did not allow us to establish the causality. Furthermore, selection bias could have been a profound problem as it was harder to get poor people and many elderly people to participate in the surveys. Also, the limitations of the WEMWBS scale can not be ruled out in this study.

## Data Availability

Click here for the data file https://individual.utoronto.ca/ahmed_3/index_files/data/data.html.

## Abbreviations

WHO: World Health Organization
STROBE: Strengthening the Reporting of Observational Studies in Epidemiology
WEMWBS: Warwick-Edinburgh Mental Well-being Scale
ANOVA: Analysis of variance
CI: Confidence interval
